# Oncolytic adenovirus coexpressing interleukin-12 and shVEGF restores antitumor immune function and enhances antitumor efficacy

**DOI:** 10.18632/oncotarget.13087

**Published:** 2016-11-04

**Authors:** Hyo Min Ahn, JinWoo Hong, Chae-Ok Yun

**Affiliations:** ^1^ Department of Bioengineering, College of Engineering, Hanyang University, Seongdong-gu, 222 Wangsimni-ro 133-791, Seoul, Korea

**Keywords:** cancer immunogene therapy, oncolytic adenovirus, interleukin-12, vascular endothelial growth factor, thymic atrophy

## Abstract

Tumor microenvironment is extremely immunosuppressive, preventing efficient induction of antitumor immunity. To overcome tumor-mediated immunosuppression and enhance the potency of immunogene therapy, oncolytic adenovirus (Ad) co-expressing interleukin (IL)-12 and vascular endothelial growth factor (VEGF)-specific short hairpin ribonucleic acid (shVEGF; RdB/IL12/shVEGF) was generated. Intratumoral injection of RdB/IL12/shVEGF induced a strong antitumor effect in an immune competent B16-F10 melanoma model. RdB/IL12/shVEGF restored immune surveillance function in tumor tissues and actively recruited immune cells by elevating the expression levels of IL-12 and interferon-γ. RdB/IL12/shVEGF efficiently suppressed expression of immunosuppressive VEGF, resulting in restoration of the antitumor immune response and prevention of thymic atrophy. *In situ* delivery of RdB/IL12/shVEGF to tumor tissues resulted in massive infiltration of differentiated CD4^+^ T cells, CD8^+^ T cells, natural killer cells, and dendritic cells to tissues surrounding the necrotic region of tumor. Furthermore, RdB/IL12/shVEGF induced a potent tumor-specific T helper type 1 immune response, implying that attenuation of the immunosuppressive environment mediated by downregulation of VEGF can significantly enhance immune stimulatory functions in the tumor milieu. Collectively, these findings indicate the potential of inducing and restoring potent antitumor immunity using intratumorally administered oncolytic Ad co-expressing IL-12 and shVEGF.

## INTRODUCTION

The field of cancer immunology and immunotherapy has been an important focus of basic and clinical research since early discoveries of tumor antigens and adaptive immunity [1–3]. Under normal physiological conditions, the immune system is capable of recognizing and eliminating tumor cells. However, tumors develop various molecular and cellular mechanisms to evade the host immune system [4]. Downregulation of immune function in cancer patients with clinically apparent tumors has been reported, resulting in poor disease management by immunotherapeutics [5]. Therefore, the success of cancer immunotherapy is critically reliant on correction of the immunosuppressive tumor microenvironment and subsequent restoration of antitumor immune function.

Interleukin (IL)-12 is a heterodimeric protein produced by activated macrophages, monocytes, dendritic cells, and stimulated B lymphocytes. IL-12 induces T helper type 1 (Th1) immunity and cytolysis by cytotoxic T-lymphocytes (CTLs) [6]. Furthermore, IL-12 stimulates production of interferon (IFN)-γ from T and natural killer (NK) cells [7]. Importantly, IL-12 has been shown to elicit potent antitumor activity in a number of *in vivo* murine tumor models by inducing an immunomodulatory effect [8–15]. However, clinical benefits of systemically administered IL-12 recombinant protein are limited by its dose-limiting side effects, poor half-life, and insufficient accumulation at tumor tissues [15–18].

Vascular endothelial growth factor (VEGF) is a pro-angiogenic protein that stimulates vasculogenesis, angiogenesis, and metastasis [19, 20]. VEGF has been shown to inhibit proliferation and maturation of T cells and dendritic cells (DCs) through interaction with T cell precursor cells in bone marrow, contributing to immunosuppression in the tumor milieu [21, 22]. Prolonged VEGF exposure has also been reported to impede thymocyte proliferation, leading to thymic atrophy in tumor-bearing mice [12]. Given the role of VEGF as an immune suppressor, we hypothesized that downregulation of VEGF expression could restore antitumor immunity through amelioration of an immunosuppressive tumor milieu and prevention of thymic atrophy.

Oncolytic adenovirus (Ad)-mediated expression of cytokine genes is a promising approach to express cytokines at high levels in tumor tissue. A clear benefit of oncolytic Ad-mediated cytokine expression system is cancer-specific amplification of therapeutic genes, because replicating viruses infect and express these genes in neighboring tumor cells. Furthermore, cytokine-expressing oncolytic Ads can elicit sustained and preferential expression of cytokines in tumor tissue, resulting in a prolonged antitumor effect and diminished toxicity compared with systemically administered recombinant cytokines [12].

In this present study, we generated oncolytic Ad co-expressing IL-12 and short-hairpin RNA-targeting VEGF (RdB/IL12/shVEGF). We hypothesized that this Ad would induce potent antitumor immunity and have good therapeutic efficacy by alleviating immunosuppression in the tumor microenvironment. Intratumoral administration of RdB/IL12/shVEGF elicited significant suppression of tumor growth in tumor-bearing immunocompetent mice. We demonstrate that the enhanced antitumor effect was associated with increased IFN-γ expression, induction of a tumor-specific immune response, recruitment of immune cells (CD4^+^, CD8^+^ T cells, DCs, and NK cells), and efficient prevention of tumor-induced thymic atrophy. Together, these results indicate that oncolytic Ad-mediated coexpression of IL-12 and shVEGF has excellent therapeutic efficacy due to restoration of antitumor immune function in tumor tissues, making this oncolytic Ad a promising candidate for potential clinical cancer immunotherapy.

## RESULTS

### Oncolytic Ad-mediated IL-12 and shVEGF expression

**Figure 1:**
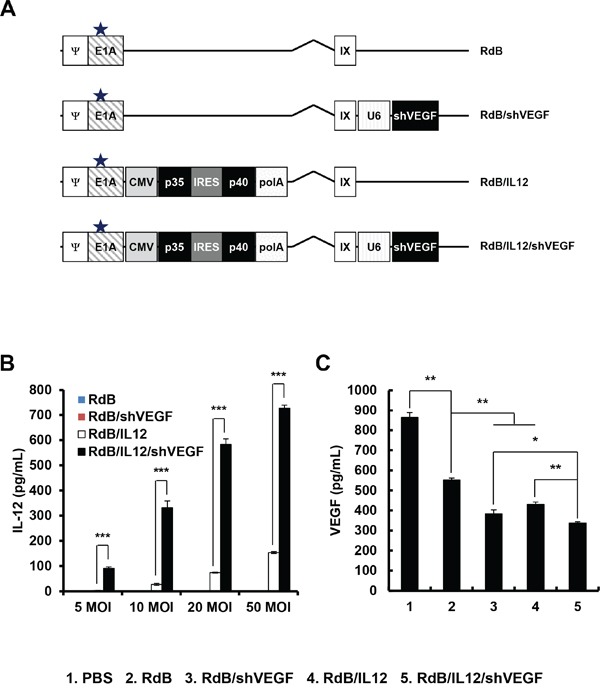
Characterization of oncolytic adenoviruses **A.** Schematic representation of the genomic structures of RdB, RdB/shVEGF, RdB/IL12, and RdB/IL12/shVEGF. RdB contains a mutated Rb protein–binding site in E1A (star), and lacks the E1B and E3 regions. Murine IL-12 and murine shVEGF were inserted into the E1 and E3 regions of the oncolytic Ad genome, respectively. **B-C.** Culture supernatants were collected at 48 hr after treatment with PBS, RdB, RdB/shVEGF, RdB/IL12, or RdB/IL12/shVEGF, and the levels of IL-12 (at 5, 10, 20, and 50 MOI) and VEGF (50 MOI) were quantified using conventional ELISA kits. *P < 0.05, **P < 0.01. Data represent the mean ± SD of triplicate experiments.

To induce efficient expression of IL-12 and long-term VEGF silencing, we generated oncolytic Ad co-expressing IL-12 and VEGF-specific shRNA (RdB /IL12/shVEGF) by inserting the murine IL-12 (p35, IRES, and p40) and shVEGF genes into the E1 and E3 regions of oncolytic Ad (RdB) [23], respectively (Figure 1A). We also generated RdB/shVEGF and RdB/IL12, which expresses either shVEGF or IL12 alone, respectively, as control oncolytic Ads.

To examine oncolytic Ad-mediated expression of IL-12 and downregulation of VEGF, B16-F10 melanoma cells were infected with RdB, RdB/shVEGF, RdB/IL12, or RdB/IL12/shVEGF. As shown in Figure 1B, RdB/IL12- or RdB/IL12/shVEGF-treated cells showed dose-dependent expression of IL-12, which was significantly higher than RdB- or RdB/shVEGF-treated cancer cells. Of interest, the level of RdB/IL12/shVEGF-mediated IL-12 expression was notably higher than that of RdB/IL12 at all viral doses (**P < 0.01), suggesting that additional expression of shVEGF in the E3 region of oncolytic Ad may positively stimulate the expression of IL-12 in a dose-dependent manner.

Furthermore, secretion level of VEGF was significantly attenuated in all oncolytic Ad-treated groups in comparison to the PBS-treated control group (Figure 1C). This in good agreement with previous reports demonstrating that Ad E1A protein downregulates VEGF expression in tumor cells [24, 25]. RdB significantly attenuated VEGF expression; VEGF secretion was 36% lower in RdB-treated cells than those treated with PBS (**P < 0.01). In addition, IL-12 has been shown to elicit an antiangiogenic effect through IFN-γ-mediated production of monokine and IFN-inducible protein 10 [26]. As expected, both RdB/IL12 and RdB/shVEGF attenuated VEGF expression by 21.9% and 38.8%, respectively, in comparison with to those treated with RdB, implying that both oncolytic Ads can efficiently express the antiangiogenic therapeutic genes IL-12 or shVEGF (**P < 0.01). Of note, RdB/IL12/shVEGF-treated cells exhibited the lowest VEGF expression level among all oncolytic Ad-treated groups (*P < 0.05 versus RdB/shVEGF, **P < 0.01 versus RdB/IL12).

To confirm the oncolytic effect of oncolytic Ads, we evaluated their ability to induce cytopathic effects in a panel of murine cancer cell lines (BNL, B16-F10, LLC, and CMT-93) and human cancer cell line (U87MG). As shown in Supplementary Figure S1, all oncolytic Ads exhibited greater cytopathic effect in U87MG than in murine cancer cells, suggesting that human cancer cells are more susceptible to oncolytic Ad infection and oncolysis. Although less susceptible to oncolytic Ads, all oncolytic Ads, including RdB without therapeutic gene, at high viral doses (MOI of 20 to 50) induced cytopathic effect in murine cancer cells, suggesting that oncolytic Ads could induce oncolysis in murine cancer cells. Importantly, RdB/IL12/shVEGF induced most potent cytopathic effect amongst the oncolytic Ads, suggesting that antiangiogenic effect of RdB/IL12/shVEGF led to potent cancer cell killing effect. Taken together, these results demonstrate that RdB/IL12/shVEGF can induce higher expression of IL-12, downregulate VEGF expression more efficiently, and elicit more potent cancer cell killing effect than either single gene-expressing RdB/IL12 or RdB/shVEGF, making it a promising platform for oncolytic Ad-mediated cancer immunogene therapy.

### Potent antitumor effect of the IL-12- and shVEGF-coexpressing oncolytic Ad in established melanoma-bearing immune-competent animals

**Figure 2:**
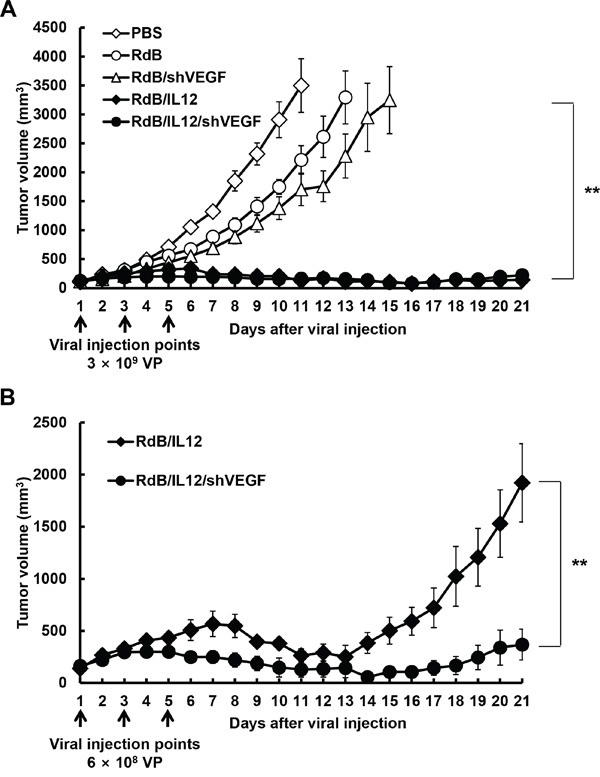
Antitumor effect of oncolytic adenoviruses in tumor-bearing mice **A.** Antitumor effect of adenovirus treatment of B16-F10 tumor-bearing C57BL/6 mice (initial tumor volume: 100 mm^3^). Mice were treated with intratumoral injections of PBS (open diamonds), RdB (open circles), RdB/shVEGF (open triangles), RdB/IL12 (filled diamonds), or RdB/IL12/shVEGF (filled circles) at 3 × 10^9^ VP. **B.** Antitumor effect of RdB/IL12 (filled diamonds) and RdB/IL12/shVEGF (filled circles) at the lower viral dose of 6 × 10^8^ VP. Tumor volume was monitored and recorded every day until the end of the study (day 21). Arrows represent injection of treatment groups. Values represent the mean ± SD (n=8). **P < 0.01.

To compare the therapeutic efficacy of oncolytic Ads expressing shVEGF, IL-12, or shVEGF plus IL-12, B16-F10 melanoma tumors established in C57BL/6 mice were injected with RdB/shVEGF, RdB/IL12, or RdB/IL12/shVEGF, along with PBS or RdB as controls. All mice in the PBS control group showed aggressive tumor growth, resulting in rapid formation of large tumors with a volume exceeding 3,000 mm^3^ by 11 days following initial treatment (Figure 2A). In contrast, RdB-, RdB/shVEGF-, RdB/IL12-, or RdB/IL12/shVEGF-treated tumors showed significantly inhibited tumor growth – there was a 36.7, 51.3, 96.4, and 95.3% reduction in tumor growth, respectively, compared to the PBS control group at 11 days after initial treatment. In particular, both IL-12-expressing oncolytic Ads (RdB/IL12 and RdB/IL12/shVEGF) elicited a significantly greater antitumor effect than RdB or RdB/shVEGF (**P < 0.01), resulting in complete remission in five of eight mice treated with RdB/IL12 or RdB/IL12/shVEGF. In marked contrast, none of the mice in the other treatment groups (PBS, RdB, and RdB/shVEGF) achieved complete remission.

Because there was no difference in therapeutic efficacy between RdB/IL12 and RdB/IL12/shVEGF at 3 × 10^9^ VP of either virus, we further assessed antitumor efficacy at a 5-fold lower viral dose (6 × 10^8^ VP). As shown in Figure 2B, tumor re-growth was observed in RdB/IL12-treated tumors on day 14 following initial oncolytic Ad administration, whereas tumor growth was still inhibited in mice treated with RdB/IL12/shVEGF with a 5.2-fold smaller tumor volume than those treated with RdB/IL12 (**P < 0.01) 21 days after the initial treatment. Taken together, these results suggest that RdB/IL12/shVEGF has prolonged and more potent therapeutic efficacy than oncolytic Ads expressing a single therapeutic gene (RdB/shVEGF or RdB/IL12).

### Increased expression of IL-12 and IFN-γ and decreased VEGF expression in tumor tissues following oncolytic Ad treatment

**Figure 3:**
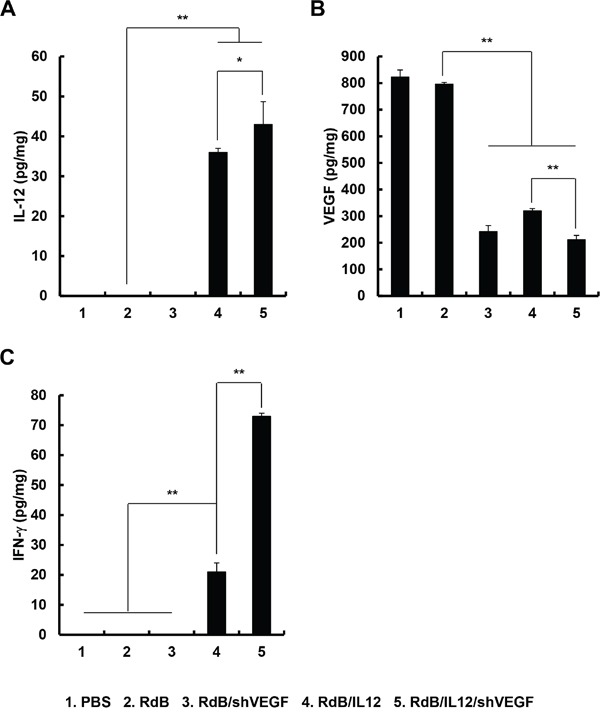
Local tumor expression of IL-12, VEGF, and IFN-γ Tumor tissues were obtained 12 days after the initial treatment with PBS, RdB (3 × 10^9^ VP), RdB/shVEGF (3 × 10^9^ VP), RdB/IL12 (6 × 10^8^ VP), or RdB/IL12/shVEGF (6 × 10^8^ VP), and ELISA was performed to estimate the levels of **A.** IL-12, **B.** VEGF, and **C.** IFN-γ in tumor tissues. Experiments were carried out in triplicate. Each data point shows the mean ± SD of IL-12, VEGF, and IFN-γ levels of the tumor cell lysates. *P < 0.05 or **P < 0.01.

To examine the levels of cytokines and VEGF produced in tumor tissues treated with RdB (3 × 10^9^ VP), RdB/shVEGF (3 × 10^9^ VP), RdB/IL12 (6 × 10^8^ VP), or RdB/IL12/shVEGF (6 × 10^8^ VP), tumor tissues were harvested 12 days after initial viral injection. As seen in Figure 3A, tumors treated with RdB/IL12 or RdB/IL12/shVEGF showed high concentrations of IL-12 (36.0 ± 1.0 or 43.0 ± 5.7 pg/mL, respectively; **P < 0.01 vs. RdB), whereas IL-12 expression was not detected in tumors treated with PBS, RdB, or RdB/shVEGF. Further, RdB/shVEGF-, RdB/IL12-, or RdB/IL12/shVEGF-treated tumors exhibited significantly lower levels of VEGF (242.5 ± 22.5, 320.6 ± 8.0, or 212.0 ± 15.7, pg/mL, respectively; **P < 0.01) than control RdB tumors (Figure 3B, 796.3 ± 6.7 pg/mL), implying that both IL12 and shVEGF can efficiently suppress VEGF expression in the tumor milieu.

IFN-γ is a cytokine that prevents tumor development and functions as an important mediator of tumor-specific immune responses [27]. Furthermore, IL-12-induced IFN-γ secretion has been reported to attenuate VEGF levels *in vivo* [28]. Therefore, we assessed the expression of IFN-γ in tumor tissues treated with various oncolytic Ads. As shown in Figure 3C, RdB/IL12- or RdB/IL12/shVEGF-treated tumors exhibited significantly higher levels of IFN-γ than RdB- or RdB/shVEGF-treated tumors (**P < 0.01). Importantly, RdB/IL12/shVEGF-treated tumors showed 3.5-fold higher IFN-γ expression than RdB/IL12-treated tumors (**P < 0.01). These results demonstrate that treatment of tumors with RdB/IL12/shVEGF tumors can induce high expression of IFN-γ and subsequently attenuate VEGF expression in tumor tissue, resulting in a shift in the T cell response toward the type 1 pattern for enhanced antitumor efficacy.

Although controlled expression of antitumor cytokines at tumor tissue can ameliorate immunosuppression and induce antitumor immune response, high cytokine levels in serum can be detrimental and cause severe side effects. Thus, we next examined whether intratumoral administration of oncolytic Ads would lead to elevated cytokine level in serum. As shown in Supplementary Figure S2, there was no detectable accumulation of both IL-12 and IFN-γ in serum following administration of oncolytic Ads (RdB, RdB/shVEGF, RdB/IL12, or RdB/IL12/shVEGF), demonstrating that oncolytic Ad-mediated elevation of antitumor cytokines was highly localized to tumor tissue.

### Generation of a tumor-specific immune response through an increase in the IFN-γ/IL-6 cytokine ratio

**Figure 4:**
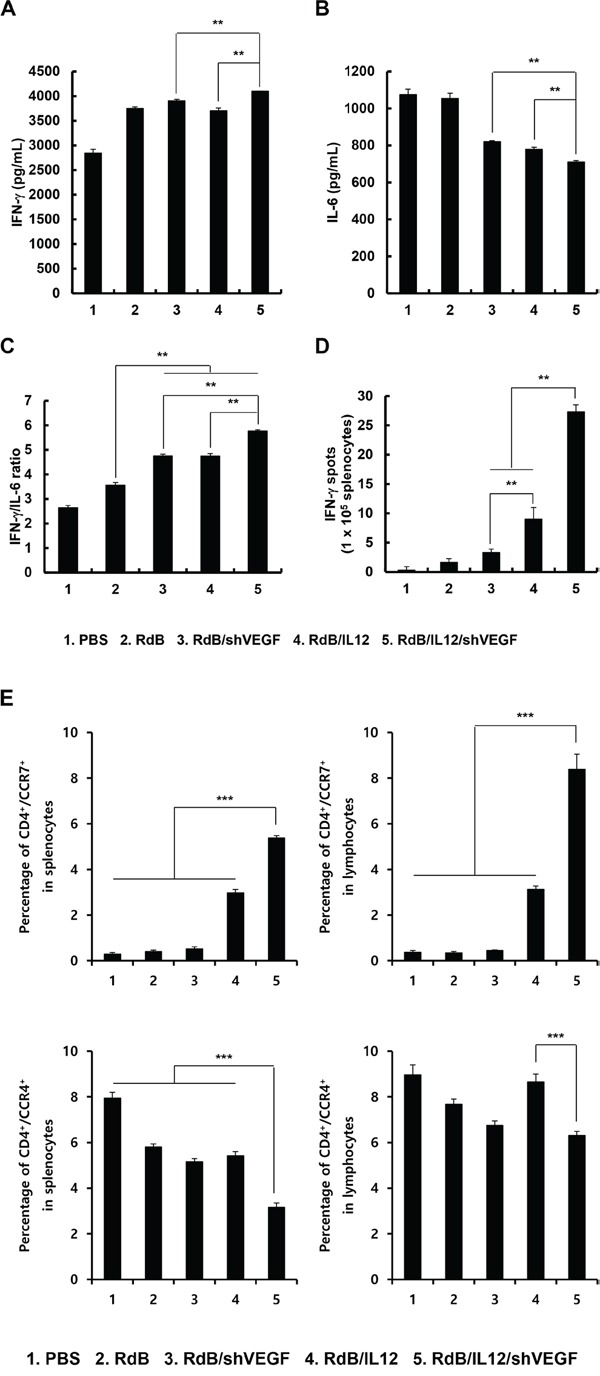
Assessment of IFN-γ/IL-6 cytokine ratio Splenocytes were harvested from mice at 12 days following the initial viral injection of PBS, RdB (3 × 10^9^ VP), RdB/shVEGF (3 × 10^9^ VP), RdB/IL12 (6 × 10^8^ VP), or RdB/IL12/shVEGF (6 × 10^8^ VP), and co-cultured with irradiated B16-F10 tumor cells for 3 days in the presence of recombinant mouse IL-2. The Th1/Th2 CBA assay was performed to determine the expression levels of IFN-γ **A.**, IL-6 **B.**, and the IFN-γ/IL-6 cytokine ratio **C.** in co-cultured supernatant. Each data point indicates the mean ± SE of triplicate experiments. **P < 0.01. **D.** Splenocytes were collected from PBS-, RdB-, RdB/shVEGF-, RdB/IL12-, or RdB/IL12/shVEGF-treated mice at 12 days following the initial viral treatment, and co-incubated with pre-irradiated B16-F10 cells for 24 hr. The IFN-γ ELISpot assay was then carried out and spots were measured with a computer-based immunospot system. Each value represents the mean spot number ± SE of triplicate experiments. **P < 0.01. **E.** spleens and DLNs were collected from mice treated with PBS, RdB, or cytokine-expressing oncolytic Ads at 12 days following the first viral treatment, and were analyzed after gating on the mononuclear lymphocyte population. CD4^+^ T cells were gated on and analyzed for CCR4^+^ or CCR7^+^ cells. Each data point indicates mean ± SD of triplicates of representative of three independent experiments. ***P < 0.001.

The shift from Th1 to Th2 cytokine expression has been shown to promote progression of malignant tumors [29, 30]. Furthermore, a strong correlation between the IFN-γ/IL-6 cytokine ratio and the therapeutic efficacy of cancer treatments has been reported [29, 30]. Therefore, we investigated whether RdB/IL12/shVEGF, which produced markedly higher amounts of Th1 cytokines (IL-12 and IFN-γ) than any other treatment group, could shift the response from a Th2 immune response to a Th1 immune response. Splenocytes of mice were harvested 12 days post initial viral injection and co-cultured with irradiated B16-F10 tumor cells for 3 days in the presence of recombinant mouse IL-2. The ratio of IFN-γ/IL-6, which corresponds to the Th1/Th2 cytokine ratio, was then analyzed in the co-cultured supernatant. As shown in Figure 4A-4C, splenocytes from RdB/IL12/shVEGF-treated mice exhibited the highest IFN-γ/IL-6 ratio among all treatment groups (**P < 0.01).

To further assess the effect of oncolytic Ads on the polarization of naïve T cells into Th1 or Th2, we evaluated T cell population in the spleen and lymph nodes following treatment with oncolytic Ads. As shown in Figure 4E, RdB/IL12/shVEGF treatment induced significantly higher accumulation of Th1 subset (CD4^+^/CCR7^+^) in the spleen and lymph node in comparison to any other treatment groups (*P* < 0.001). In addition, immunosuppressive Th2 population (CD4^+^/CCR4^+^) was significantly lower in RdB/IL12/shVEGF-treated group in comparison to any other treatment groups (*P <* 0.001). Together, these results demonstrate that efficient secretion of IL-12 and downregulation of VEGF by RdB/IL12/shVEGF can induce polarization of naïve CD4^+^ T cells toward Th1 and conjunctively inhibit transition into immunosuppressive Th2 subset by increasing IFN-γ expression while suppressing IL-6 expression.

To assess tumor-specific immune response, splenocytes from mice treated with RdB, RdB/shVEGF, RdB/IL12, or RdB/IL12/shVEGF were further assessed for tumor cell-specific IFN-γ-secreting lymphocytes by IFN-γ ELISpot assay. As shown in Figure 4D, the frequency of IFN-γ-secreting immune cells recovered from mice injected with RdB/IL12 was greater than that from mice treated with RdB/shVEGF (**P < 0.01), and a significantly greater number of IFN-γ-secreting immune cells was observed in the RdB/IL12/shVEGF-treated group than the RdB/IL12-treated group (**P < 0.01), suggesting that expression of IL-12 is more critical for induction of the Th1 immune responses than downregulation of VEGF alone. Importantly, RdB/IL12/shVEGF-treated group had significantly more IFN-γ-secreting immune cells than the groups treated with RdB/IL12 or RdB/shVEGF, indicating that the co-expression of IL-12 and shVEGF can induce a potent Th1-type immune response. Taken together, these results indicate that the observed potent antitumor efficacy of RdB/IL12/shVEGF in Figure 3 was most likely due to induction of a tumor-specific immune response.

### Abundant infiltration of CD4^+^ T cells, CD8^+^ T cells, DCs, and NKs in RdB/IL12/shVEGF-treated tumor tissues

**Figure 5:**
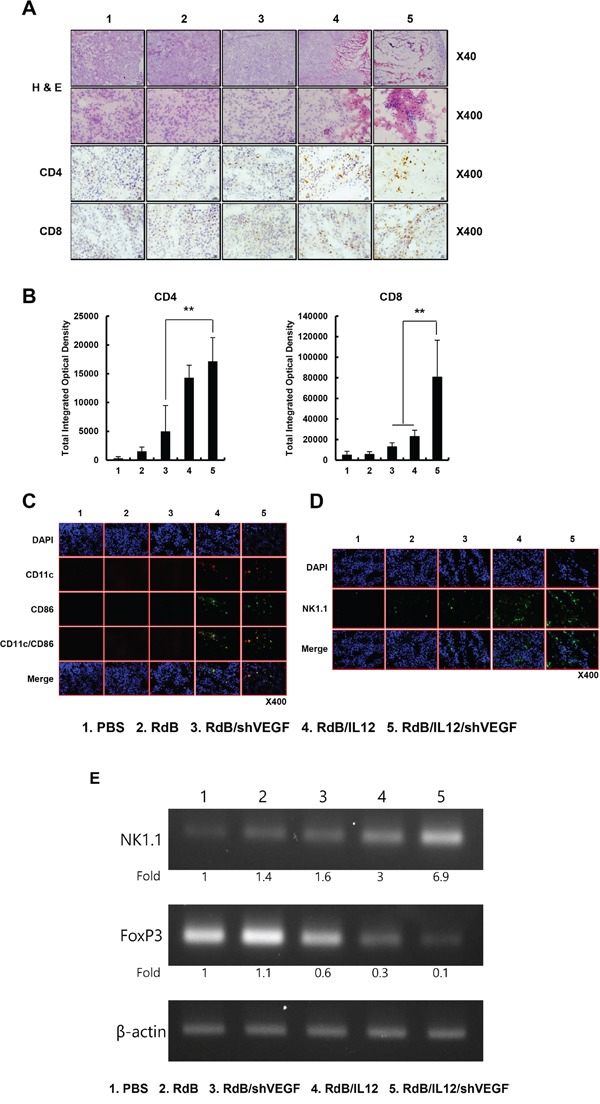
Histological and immunohistochemical analysis Oncolytic Ads were injected PBS, RdB (3 × 10^9^ VP), RdB/shVEGF (3 × 10^9^ VP), RdB/IL12 (6 × 10^8^ VP), or RdB/IL12/shVEGF (6 × 10^8^ VP) on days 1, 3 and 5, and tumors were collected at day 12 following the initial viral injection for histological and immunohistochemical analysis. **A.** Paraffin sections of tumor tissue were stained with H & E. Original magnification: ×40 and ×400. Cryosections of tumor tissue were stained with anti-CD4 or anti-CD8 monoclonal antibody. Original magnification: ×400. **B.** The numbers of CD4^+^ T and CD8^+^ T cells were semi-quantitatively measured with MetaMorph® image analysis software (**P < 0.01). **C.** Cryosections of tumor tissue were stained with anti-CD11c or anti-CD86 monoclonal antibody. Original magnification: ×400. **D.** Cryosections of tumor tissue stained with anti-NK-1.1 monoclonal antibody. Original magnification: ×400. **E.** The level of FoxP3 and NK1.1 was quantified by conventional RT-PCR assay in tumor lysate, and the expression levels of FoxP3 and NK1.1 were semi-quantitatively analyzed using ImageJ software (National Institutes of Health, Bethesda, MD).

Histological analysis revealed markedly enhanced tumor necrosis in tumor tissues treated with RdB/IL12/shVEGF in comparison with RdB-, RdB/shVEGF- or RdB/IL12-treated tumor tissues (Figure 5A). CD4^+^ T and CD8^+^ T cells are known to play a central role in regulating antigen-specific immune responses, including those mediated by tumor-associated antigens [31]. Higher frequencies of CD4^+^ T and CD8^+^ T cells were observed in the tumors treated with RdB/IL12/shVEGF in comparison to RdB-, RdB/shVEGF-, or RdB/IL12-treated tumor tissues (Figures 5A and 5B), indicating that RdB/IL12/shVEGF induced the strongest activation and recruitment of T cells to the tumor tissues. Furthermore, immunofluorescence staining of tumor tissues with CD11c-, CD86-, and NK-1.1-specific Abs revealed that RdB/IL12/shVEGF induced the greatest infiltration of immune cells (DC and NK) into the tumor tissues (Figures 5C and 5D). Importantly, RdB/IL12/shVEGF-treated tumor tissues exhibited markedly higher co-localization of CD86^+^/CD11c^+^ DCs than the other treatment groups. These results suggest that RdB/IL12/shVEGF can enhance DC activation and infiltration in tumor tissue, indicating effective induction of adaptive immunity against cancer. Reverse transcription polymerase chain reaction (RT-PCR) analysis of tumor lysates with NK cell marker further supported the notion that RdB/IL12/shVEGF treatment induced greatest infiltration of NK cells (Figure 5E). Of interest, RdB/IL12/shVEGF treatment resulted in lowest accumulation of immunosuppressive regulatory T cells (Treg; FoxP3-positive), suggesting that RdB/IL12/shVEGF can reverse tumor-induced immunosuppression to enhance intratumoral infiltration of immune cells. Taken together, these results suggest that the strong antitumor effect elicited by RdB/IL12/shVEGF is associated with markedly enhanced infiltration and activation of CD4^+^ T, CD8^+^ T, DC, and NK cells to tumor tissue and concurrent inhibition of immunosuppressive Treg accumulation.

### Prevention of tumor-induced thymic atrophy by RdB/IL12/shVEGF treatment

**Figure 6:**
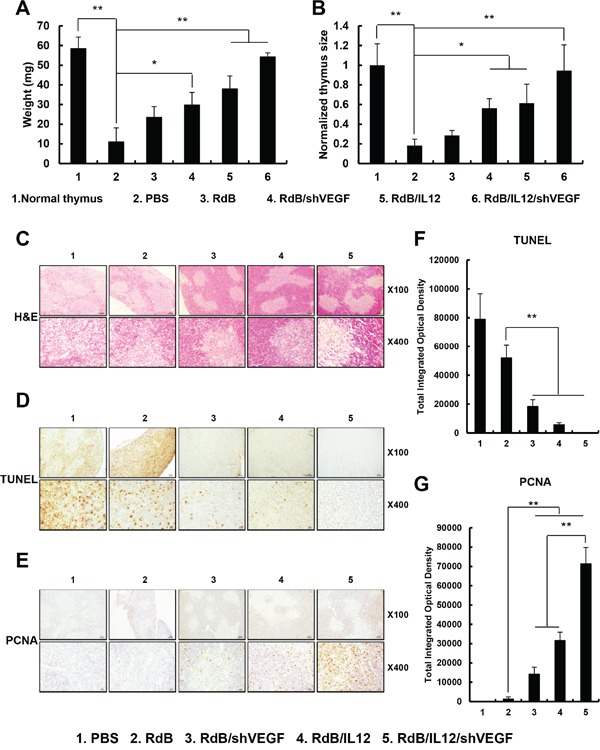
Prevention of thymic atrophy **A, B.** The weight and size of thymuses in mice treated with PBS, RdB (3 × 10^9^VP), RdB/shVEGF (3 × 10^9^ VP), RdB/IL12 (6 × 10^8^ VP), or RdB/IL12/shVEGF (6 × 10^8^ VP) **C.** Sections from paraffin-embedded thymuses were stained with H & E. Original magnification: ×40 and ×400. **D.** Detection of apoptosis in thymus tissue by TUNEL assay. Original magnification: ×40 and ×400. **E.** PCNA staining was performed to assess thymocyte proliferation. Original magnification: ×40 and ×400. **F, G.** Semi-quantitative assessment of TUNEL assay and PCNA staining results using MetaMorph® image analysis software. **P < 0.01.

Previous studies reported that thymic atrophy observed in tumor-bearing mice contributed to suppression of host immunity against the tumor [32, 33]. Furthermore, it has been shown that prolonged exposure to recombinant VEGF induces thymic atrophy *in vivo* [21]. These findings suggest that prevention of thymic atrophy could alleviate tumor-induced immunosuppression. Therefore, we first examined the effects of the various oncolytic Ads (RdB, RdB/shVEGF, RdB/Il12, or RdB/IL12/shVEGF) on prevention of thymic atrophy through observation of thymus weight and size in tumor-bearing mice. As shown in Figures 6A-6B and Supplementary Figure S4, we observed thymic atrophy in mice treated with PBS; the thymuses of these mice were significantly lighter and smaller than normal thymuses (**P < 0.01). The weight and size of thymuses were significantly higher in tumor-bearing mice treated with RdB/shVEGF, RdB/IL12, or RdB/IL12/shVEGF than PBS-treated mice, suggesting that oncolytic Ad treatment could prevent thymic atrophy (**P < 0.01, *P < 0.05). These results were proportionally correlated with the VEGF suppression data shown in Figure 1C. Importantly, mice treated with RdB/IL12/shVEGF had thymuses of similar size and weight to those of normal thymuses, suggesting that combinatorial expression of Ad E1A, IL12, and shVEGF could efficiently prevent thymic atrophy.

To further assess morphologic and biologic changes in the thymus, we performed histological and immunohistochemical staining of thymic tissue collected from tumor-bearing mice treated with PBS (control) or various oncolytic Ads. We observed clearly segregated areas between the cortex and medulla zones in thymus tissues from tumor-bearing mice treated with RdB/shVEGF-, RdB/IL12-, or RdB/IL12/shVEGF, whereas the thymic structure was abnormally disrupted in mice treated with PBS or RdB on day 12 following initial treatment (Figure 6C). These results suggest that both shVEGF and IL-12 expression can efficiently preserve the structural integrity of the thymus and prevent tumor-induced atrophy. To investigate the mechanisms behind the prevention of thymic atrophy mediated by shVEGF and IL-12, the effect of each treatment on apoptosis and proliferation of thymocytes was assessed. PBS- or RdB-treated mice showed more extensive apoptosis in thymic tissues than those treated with RdB/shVEGF, RdB/IL12, or RdB/IL12/shVEGF, indicating that expression of either shVEGF or IL-12 can reduce the induction of apoptosis in thymic tissues (Figures 6D and 6F, **P < 0.01). Additionally, RdB/shVEGF-, RdB/IL12-, or RdB/IL12/shVEGF-treated mice had higher populations of thymocytes undergoing active proliferation than RdB-treated mice (Figures 6E and 6G, **P < 0.01), which inversely correlates with the TUNEL data. Of note, RdB/IL12/shVEGF-treated thymuses had similar morphological attributes to the thymuses of healthy mice without tumors, suggesting that co-expression of IL-12 and shVEGF can alleviate thymic atrophy. Overall, these results demonstrate that RdB/IL12/shVEGF can effectively prevent thymic atrophy in tumor-bearing mice via the inhibition of apoptosis and induction of proliferation in the thymus.

## DISCUSSION

Weakened immune function in cancer patients is well-characterized [5]. One of the immunoescape mechanisms mediated by tumors is production of soluble immune suppressors, such as IL-10, TNF, TGF-β, and VEGF [34]. In particular, clinical data indicate that VEGF inhibits DC differentiation, and its expression level is inversely correlated with DC number in tumor tissue and peripheral blood of cancer patients [22, 35, 36]. Given the critical role of DCs as a crucial component of the adaptive immune response, we hypothesized that suppression of VEGF in the tumor microenvironment would restore the host's immune surveillance activity against tumors.

IL-12 facilitates Th1 differentiation and augments the cytolytic effect of NK cells and CTL, resulting in enhanced antitumor immunity [37, 38]. Importantly, IL-12 treatment has shown to attenuate VEGF expression, tumor vascularization, and increase apoptosis in tumor tissues [39]. These attributes of IL-12 makes it a good candidate for combined use with a VEGF-specific shRNA system as both can induce a potent antitumor immune response through downregulation of immunosuppressive VEGF.

Recombinant cytokines have been reported to induce potent immunomodulation in tumor-bearing mice, resulting in potent antitumor efficacy [40]. However, systemic treatment of tumor-bearing animals and cancer patients with cytokines at therapeutic dose can cause severe side effects [41–44]. Furthermore, local injection of recombinant cytokines is ineffective due to the short half-life and rapid diffusion of cytokines from tumor tissue [45]. In this regard, oncolytic Ad-mediated cytokine expression in tumor tissue could potentially induce potent antitumor immunity without severe aberrant effects. Oncolytic Ads can preferentially express cytokines at high levels for prolonged periods of time in tumor tissue, leading to prolonged induction of antitumor immunity and attenuated toxicity in comparison with systemically administered recombinant cytokines [8, 9, 12, 46].

In this present report, we generated an oncolytic Ad co-expressing IL-12 and shVEGF (RdB/IL12/shVEGF) to ameliorate the immunosuppressive tumor microenvironment and cytokine-mediated induction of antitumor immunity. Cancer cells infected with RdB/IL12/shVEGF showed significantly higher IL-12 expression than those infected with RdB/IL12 (Figure 1B), implying that an additional expression of shVEGF in the E3 region of Ad may increase the expression of IL-12. This is in good agreement with our previous reports where insertion of additional transgene in the E1 or E3 region of Ad genome either attenuate or enhance expression of the transgene in the other region [9, 10, 12]. Furthermore, RdB/IL12/shVEGF-treated cancer cells exhibited the lowest VEGF expression among all treatment groups (Figure 1C), indicating that higher IL-12 expression in combination with co-expression of shVEGF can downregulate VEGF expression more effectively than control oncolytic Ads expressing single or no therapeutic genes (RdB, RdB/IL12, or RdB/shVEGF).

An aberrant Th1/Th2 cytokine balance, in which Th2 cytokines are overexpressed, has been shown to promote progression of cancers in patients, and a strong correlation between Th1/Th2 cytokine balance and the efficacy of cancer treatments has been reported [29, 30]. IFN-γ, a Th1 cytokine, plays an important role in the induction of innate and adaptive immune responses against cancer [47], making IFN-γ a key mediator of the antitumor-immune response. We observed significantly augmented expression levels of IFN-γ from RdB/IL12/shVEGF-treated mice in comparison to RdB/IL12, implying that additional expression of shVEGF might enhance antitumor immunity by more potently downregulating VEGF expression (Figure 3C). These findings suggest that RdB/IL12/shVEGF can alleviate tumor-mediated immunosuppression and induce potent antitumor immunity through stimulating expression of IFN-γ and inducing Th1 immunity, which has been previously reported to induce antitumor immunity by activating CTLs, NK, and Th1 cells as well as increasing cancer cell immunogenicity through enhancing antigen presentation [48]. Efficient restoration of antitumor immunity in tumor tissue by treatment with RdB/IL12/shVEGF was further supported by the significantly higher IFN-γ/IL-6 cytokine ratio in tumor tissues treated with RdB/IL12/shVEGF than the other treatment groups, indicating efficient polarization of T cell responses toward the type 1 pattern and the establishment of a tumor microenvironment more favorable to the activation of tumor-specific immune cells (Figures 4A, 4B, and 4C). Importantly, the IFN-γ-secreting cell population was significantly greater in mice treated with RdB/IL12/shVEGF than mice treated with RdB/IL12, indicating that downregulation of VEGF expression can function as a potent adjuvant for IL-12-mediated induction of Th1 immunity in the tumor milieu. Taken together, these results demonstrate that the therapeutic mechanism of RdB/IL12/shVEGF involves reversal of immunosuppressive Th2 immunity toward Th1 immunity and the recruitment of tumor-specific IFN-γ-producing T cells in tumor tissues.

Subsequent experiments demonstrated that the enhanced antitumor effect was caused by abundant infiltration of CD4^+^T cell, CD8^+^ T cell, and NK cells (Figures 5A, 5B, and 5D), and efficient downregulation of immunosuppressive Treg cell population (Figure 5E). In strong agreement with these results, treatment with RdB/IL12/shVEGF significantly increased the number of CD86^+^/CD11c^+^ mature DCs that infiltrated the tumor tissue in comparison with either RdB/shVEGF or RdB/IL12, suggesting that RdB/IL12/shVEGF makes the tumor microenvironment more favorable for recruitment and infiltration of DCs (Figure 5C).

The thymus is a specialized primary lymphoid organ of the immune system that functions well into adult life to produce T lymphocytes, and also functions as a major site of T cell maturation [32, 33]. The thymus is composed of two identical lobes; each lobe of the thymus can be divided into a central medulla and a peripheral cortex which is surrounded by an outer capsule [49]. The cortex is the location of the earliest events in thymocyte development, where T cell receptor gene rearrangement and positive selection take place. The medulla is where latter events in thymocyte development occur. The medulla is specialized to allow thymocytes to undergo additional rounds of negative selection to remove auto-reactive T cells from the mature repertoire [50]. Previous studies have reported that thymic atrophy occurs in tumor-bearing mice [32, 33], which can suppress T cell development and T cell-mediated immunity [51]. Elevated VEGF expression has been speculated to cause thymic atrophy in tumor-bearing mice and tumor-mediated immunosuppression [21]. We previously reported that IL-12 plays an important role in the prevention of thymic atrophy in tumor-bearing mice [12]. In agreement with these previous studies, thymic atrophy in tumor-bearing mice was effectively prevented by treatment of the mice with RdB/IL12/shVEGF; reduction of apoptosis and increased proliferation of thymocytes were evident when compared with the other treatment groups (Figure 6). Thymus weight and size were inversely correlated with the tumor burden data shown in Figure 2, suggesting that oncolytic Ad-mediated restoration of immune function in the tumor milieu and attenuation of tumor burden can prevent thymic atrophy-mediated immunosuppression in tumor tissues.

## MATERIALS AND METHODS

### Cell lines and cell culture

Dulbecco's modified Eagle's medium (DMEM; GIBCO, Grand Island, NY, USA) supplemented with 10% fetal bovine serum (FBS; GIBCO), L-glutamine (2 mmol/L), penicillin (100 IU/mL), and streptomycin (50 mg/mL) was used as the culture medium. HEK293 (human embryonic kidney cell line expressing the adenoviral E1 region), U87MG (human glioma cell line), BNL (murine liver cancer cell line), B16-F10 (murine melanoma cell line), LLC (murine lung cancer cell line), and CMT-93 (murine polyploid carcinoma cell line) were obtained from the American Type Culture Collection (ATCC, Manassas, VA). All cell lines tested negative for *Mycoplasma* when tested by Hoeschst dye (MP Biomedicals, Irvine, CA, USA) staining, cell culture, and polymerase chain reaction.

### Animal studies

Six- to eight-week-old male C57BL/6 mice were purchased from Orient Bio Inc. (Sungnam, Korea) and maintained in a laminar air-flow cabinet under a specific pathogen-free environment. All facilities were approved by the AAALAC (Association for Assessment and Accreditation of Laboratory Animal Care). All animal experiments were conducted according to the institutional guidelines established by the Hanyang University Institutional Animal Care and Use Committee.

### Generation of oncolytic Ad expressing IL-12 and shVEGF

Target siRNA sequences for VEGF were selected using a dedicated program provided by Ambion Inc. (Austin, TX, USA). Double-stranded RNA oligonucleotides corresponding to nucleotides 92-112 of murine VEGF mRNA (GenBank accession number gi: 70608153) were synthesized using a Silencer™ siRNA construction kit (Ambion) [52]. To generate shRNA targeting murine VEGF, a DNA fragment that would express shRNA targeting positions 92-112 of murine VEGF was generated by annealing the sense oligonucleotide 5’-gatcccggAAGGAGAGCAGAAGTCCCATGttcaagagaCATGGGACTTCTGCTCTCCTTtttttttg gaaa-3’ and its cognate antisense oligonucleotide 5’-tttccaaaaaaaAAGGAGAGCAGAAGTCCCATGtctcttgaaCATGGGACTTCTGCTCTCCTTccgggatc-3’; the 21 nucleotide VEGF target sequence is underlined and in uppercase letters, whereas the nine nucleotide-long hairpin and the sequence required for directional cloning are depicted in lowercase letters. The shVEGF gene fragment was then inserted into pSP72-E3/U6 [24], resulting in pSP72-E3/U6-shVEGF. To generate an oncolytic Ad expressing shVEGF at the E3 region, pSP72-E3/U6-shVEGF was co-transformed with the Ad total vector pRdB [23] at the E3 region into *Escherichia coli* BJ5183 for homologous recombination, generating the pRdB/shVEGF Ad vector (Figure 1A). To construct an oncolytic Ad expressing IL-12 and shVEGF at the E1 and E3 regions, respectively, pSP72-E3/U6-shVEGF was co-transformed with the Ad total vector pRdB/IL12 [12] at the E3 region into *Escherichia coli* BJ5183 for homologous recombination, generating the pRdB/IL12/shVEGF Ad vector (Figure 1A). As control oncolytic Ads, we also amplified RdB/shVEGF and RdB/IL12 [12], which express either shVEGF or IL12 alone, respectively. All viruses were propagated using HEK293 cells, and purification, titration, and quality analysis of all Ads were carried out as described previously [11, 12].

### Enzyme-linked immunosorbent assay for IL-12 and VEGF expression

B16-F10 melanoma cells were plated onto six-well plates at 3 × 10^5^ cells per well, and then infected with RdB, RdB/shVEGF, RdB/IL12, or RdB/IL12/shVEGF at 50 MOI. At 48 hr after infection, the supernatants were collected. Levels of IL-12 and shVEGF were determined by ELISA according to the manufacturer's instructions (IL-12 ELISA kit: Endogen, Woburn, MA, USA; VEGF ELISA kit: R&D Systems, Minneapolis, MN, USA).

### *In vivo* antitumor effect

B16-F10 cells (5 × 10^5^) were injected subcutaneously into the right abdomen of 6- to 7-week-old male C57BL/6 mice. When the tumor volume reached around 100 mm^3^, mice were sorted into groups with similar mean tumor volumes, and different doses of oncolytic Ads suspended in a total volume of 50 μl PBS (RdB or RdB/shVEGF at 3 × 10^9^ VP; RdB/IL-12 or RdB/IL-12/shVEGF at 3 × 10^9^ or 6 × 10^8^ VP per tumor) were injected intratumorally three times every other day. Tumor growth was monitored every day by measuring two perpendicular tumor diameters using electronic calipers (Fowler, Inc., Switzerland). Tumor volume was calculated using the following formula: volume = 0.523*L* (*W*)^2^, where *L* is length and *W* is width.

### Quantification of cytokines in tumor tissue and IFN-γ/IL-6 ratio in splenocytes

Tumor tissues were collected from PBS-, RdB-, RdB/shVEGF-, RdB/IL12-, or RdB/IL12/shVEGF-treated mice at 12 days after the initial treatment. Tumor tissues were homogenized (ART-MICCRA D-8; ART Moderne Labortechnik, Germany) in ice-cold radioimmunoprecipitation assay buffer (Elipis Biotech, South Korea) with a proteinase inhibitor cocktail (Sigma, St. Louis, MO, USA). Levels of IL-12, VEGF, and IFN-γ were measured by conventional ELISA kits (IL-12 ELISA kit: Endogen, VEGF ELISA kit: R&D Systems and IFN-γ ELISA kit: Endogen). In addition, Th1/Th2 type cytokine expression profiles from supernatant co-cultured with splenocytes and irradiated B16-F10 (5,000 rad) were estimated using a Th1/Th2 CBA kit (BD Biosciences Pharmingen, San Diego, CA, USA).

### IFN-γ enzyme-linked immune spot assay in splenocytes

At 7 days following treatment with PBS, RdB, RdB/shVEGF, RdB/IL12, or RdB/IL12/shVEGF, spleens were obtained aseptically from tumor-bearing mice, and unicellular splenocytes were prepared as described previously [8]. Prepared spleen cells were co-cultured with irradiated B16-F10 (5,000 rad) tumor cells for 3 days in the presence of recombinant mouse IL-2 (100 U/mL; R&D Systems). An IFN-γ ELISpot assay was then carried out as described previously [8]. Spots were measured using a computer-based immunospot system (AID ELISpot Reader System version 3.4; Autoimmun Diagnostika GmbH, Germany).

### Fluorescence-activated cell sorting (FACS) analysis

Spleen and draining lymph nodes (DLNs) were harvested at 12 days following first viral inoculation of the B16-F10 tumor-bearing mice. Before staining, cells were treated with saturating anti-CD16/CD32 (Biolegend, San Diego, CA) in staining buffer (2% FBS and 0.02% sodium azide in PBS). Cells were stained with fluorescein isothiocyanate (FITC)-conjugated anti-CD4 (eBioscience), percp-CY5.5-conjugated anti-CD4 (BD Biosciences Pharmingen), PE-CY7-conjugated anti-CCR4 (Biolegend), and/or PE-conjugated anti-CCR7 (Biolegend). To determine the percentage of CCR4- or CCR7-expressing CD4^+^ T cell population, splenocytes or lymphocytes were gated by plotting forward vs. side scatter followed by gating on CD4^+^ cells. Gated cells were then analyzed for CCR4^+^ or CCR7^+^ cells. Samples were analyzed using BD Biosciences BD-LSR II Analytic Flow Cytometer and FACSDiva software (BD Biosciences Pharmingen).

### Histology and immunohistochemistry

Tumor or thymus tissues collected from tumor-bearing mice treated with PBS, RdB, RdB/shVEGF, RdB/IL12, or RdB/IL12/shVEGF were fixed in 10% neutral buffered formalin (Junsei Chemical, Japan), embedded in paraffin, and cut into 4-μm-thick sections. Representative sections were stained with H & E, and then examined by light microscopy. To detect lymphocytes and DCs, tumor tissues were frozen in Optimal Cutting Temperature compound (Sakura Finetec, Torrance, CA, USA) and cut into 10-μm-thick sections. The cryosections were incubated with rat anti-mouse CD4 monoclonal Ab (BD Biosciences Pharmingen), rat anti-mouse CD8 monoclonal Ab (BD Biosciences Pharmingen), or mouse anti-mouse PCNA monoclonal Ab (DAKO, Denmark) as the primary Ab, and then treated with horseradish peroxidase-conjugated goat anti-rat IgG (BD Biosciences Pharmingen) or horseradish peroxidase-conjugated goat anti-mouse IgG (Southern Biotech, Birmingham, AL, USA) as the secondary Ab. Diaminobenzidine/hydrogen peroxidase (DAKO) was used as the chromogen substrate. All slides were counterstained with Meyer's hematoxylin (Sigma). The expression levels of CD4, CD8, and PCNA were semi-quantitatively analyzed using MetaMorph^®^ image analysis software (Universal Image Corp., UK). Results are expressed as the mean optical density of five different digital images.

### Immunofluorescence staining

For immunofluorescence staining of CD11c, CD86, and NK-1.1 cells, cryosections were treated with hamster anti-mouse CD11c monoclonal Ab (BD Biosciences Pharmingen), rat anti-mouse CD86 monoclonal Ab (BD Biosciences Pharmingen), or mouse anti-mouse NK-1.1 monoclonal Ab (BioLegend, San Diego, CA, USA) and incubated overnight at 4°C, and then treated with Alexa Fluor® 568 (Red)-labeled goat anti-hamster IgG (Invitrogen, Carlsbad, CA, USA), Alexa Fluor® 488 (Green)-labeled goat anti-rat IgG (Invitrogen), or Alexa Fluor® (Green)-labeled goat anti-mouse IgG (Invitrogen) at room temperature for 1 hr, respectively. For counterstaining, the samples were incubated with 4,6-diamidino-2-phenylindole (Sigma). Slides were mounted with Vectashield mounting medium (Vector Laboratories, Burlingame, CA, USA) and cells were viewed under a confocal laser-scanning microscope (LSM510, Carl Zeiss MicroImaging, Thornwood, NY, USA).

### Terminal deoxynucleotidyl transferase dUTP nick end labeling assay (TUNEL)

The apoptotic thymocyte population was assessed by TUNEL assay as described previously [53]. Apoptotic cells were visually identified in five randomly selected fields and photographed at magnifications of ×100 and ×400. The level of apoptotic thymocytes was semi-quantitatively analyzed using MetaMorph^®^ image analysis software (Universal Image Corp). Results are expressed as the mean optical density of five different digital images.

### Harvest and weigh of thymuses

On day 12 after initial viral treatment, all thymuses were removed and immediately placed in Roswell Park Memorial Institute 1640 medium (GIBCO), and the weight of each thymus was determined using an electronic scale (Ohaus Corp., Florham Park, NJ, USA). The size of the thymus was semi-quantitatively analyzed using ImageJ software (National Institutes of Health, Bethesda, MD, USA).

### Statistical analysis

Data are expressed as means ± standard deviation (SD). Statistical comparisons were made using Stat View software (Abacus Concepts, Inc., Berkeley, CA, USA) and the Mann-Whitney test (non-parametric rank sum test). *P* values less than 0.05 were considered statistically significant (*P < 0.05, **P < 0.01, *** *P* < 0.001).

## SUPPLEMENTARY MATERIALS FIGURES


