# Supplementary material for: Oncolytic adenovirus coexpressing interleukin-12 and shVEGF restores antitumor immune function and enhances antitumor efficacy
Source: Oncotarget. 2016 Nov 4;7(51):84965–80. doi: 10.18632/oncotarget.13087 (PMC5356712; doi:10.18632/oncotarget.13087)
Supplement: Supplementary file 1 [file oncotarget-07-84965-s001.pdf]

Oncolytic adenovirus coexpressing interleukin-12 and shVEGF restores antitumor immune function and enhances antitumor efficacy

SUPPLEMENTARY FIGURES

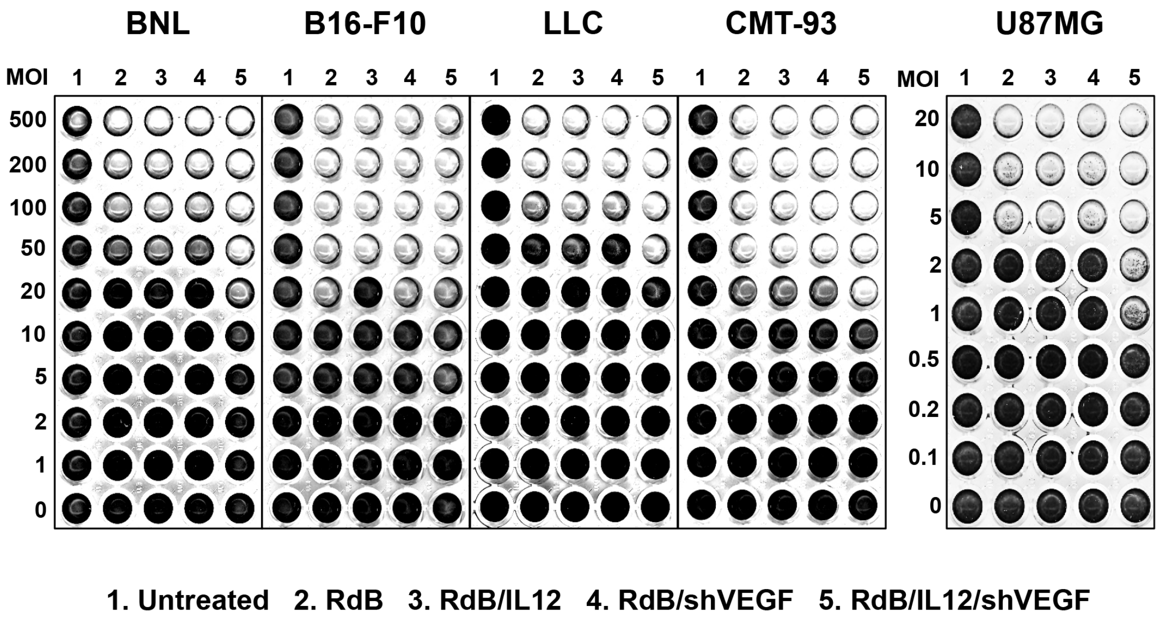

Supplementary Figure S1: Cytopathic effect of oncolytic Ads expressing IL-12 and/or shVEGF in four different murine cancer cell lines: BNL, B16-F10, LLC, and CMT-93, and human cancer cell line: U87MG. Similar results were obtained from at least three separate experiments.

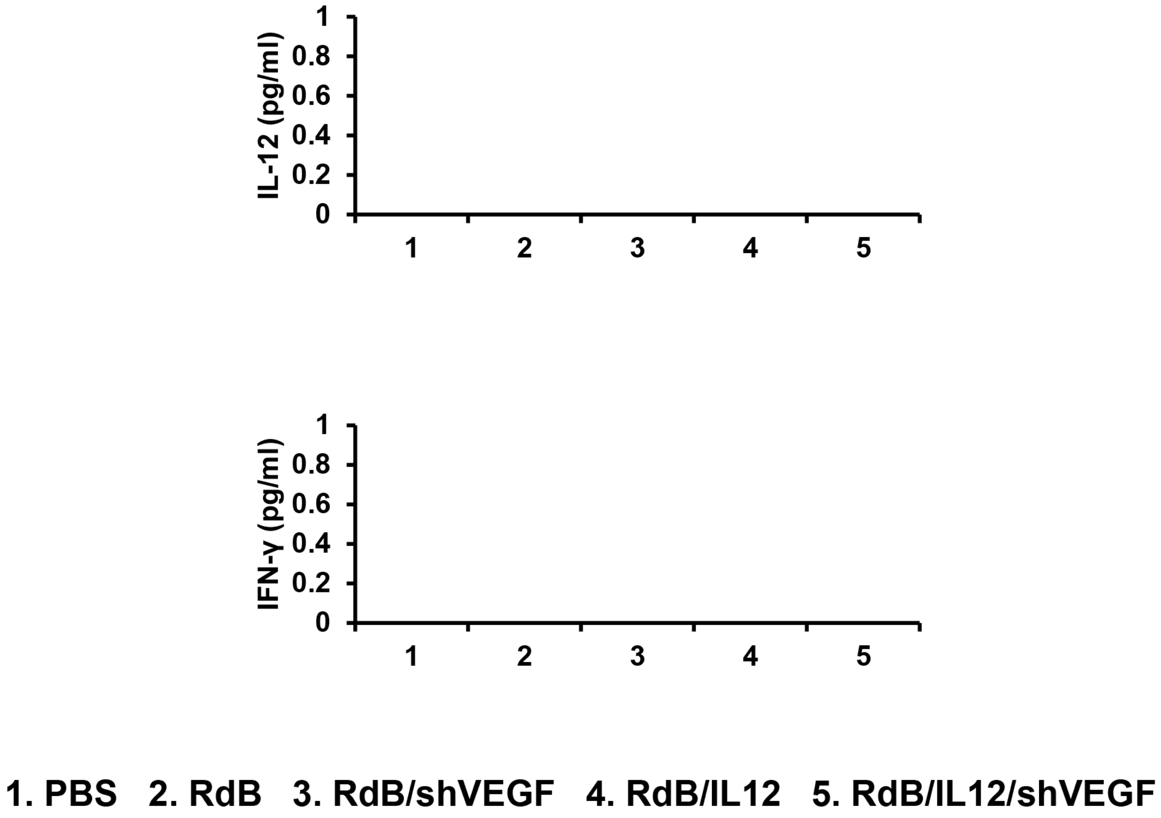

Supplementary Figure S2: Oncolytic Ads were injected PBS, RdB (3×10^9 VP), RdB/shVEGF (3×10^9 VP), RdB/IL12 (6×10^8 VP), or RdB/IL12/shVEGF (6×10^8 VP) on days 1, 3 and 5, and serums were collected at day 12 following the initial viral injection, and the level of IL-12 and IFN-γ.

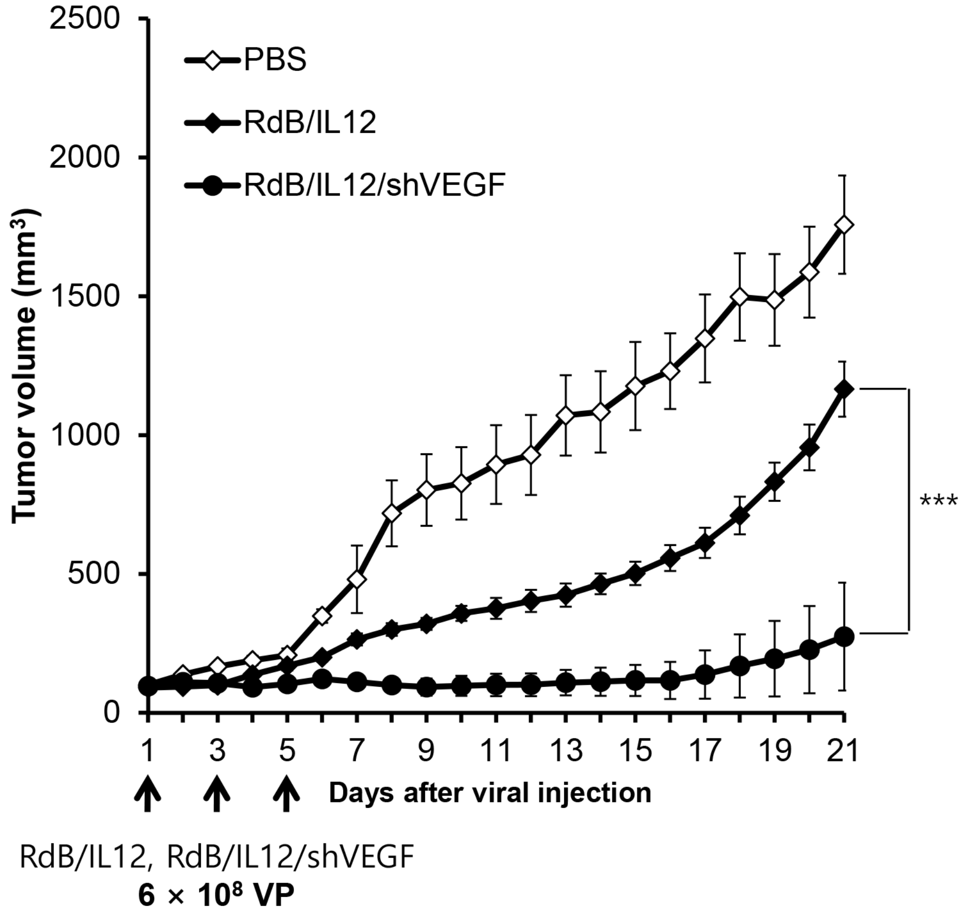

Supplementary Figure S3: Antitumor effect of oncolytic Ads in RENCA tumor-bearing BALB/C mice (initial tumor volume: 100 mm^3). Mice were intratumorally injected with 6 × 10^8 VP of RdB/IL12 (filled diamonds) or RdB/IL12/shVEGF (filled circles), along with PBS as control (open diamonds).

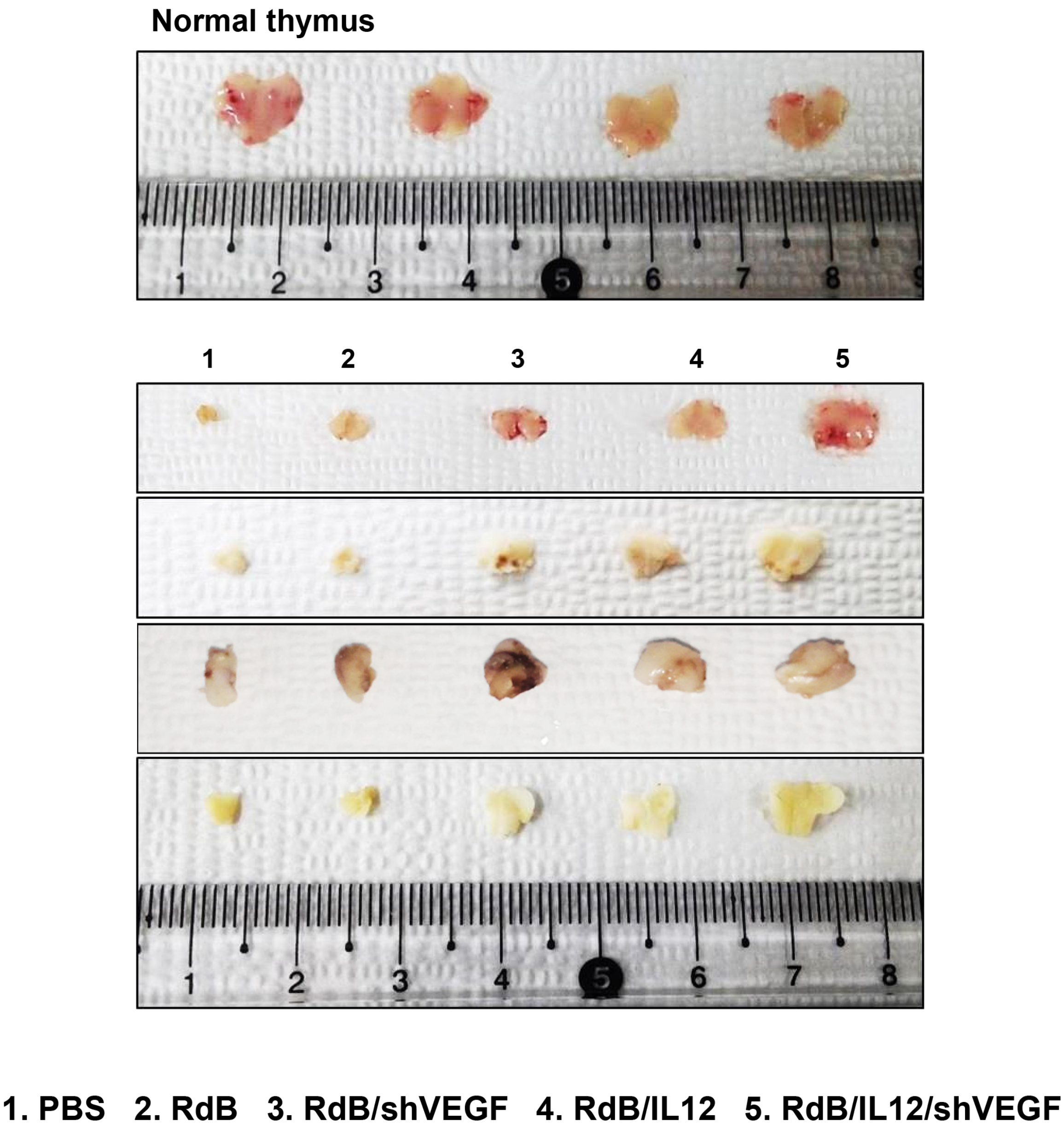

Supplementary Figure S4: Images of whole thymuses from non-tumor-bearing mice (normal mice) or PBS-, RdB-, RdB/shVEGF-, RdB/IL12-, or RdB/IL12/shVEGF-treated tumor-bearing mice.
